# The role of microbial amyloid in neurodegeneration

**DOI:** 10.1371/journal.ppat.1006654

**Published:** 2017-12-21

**Authors:** Robert P. Friedland, Matthew R. Chapman

**Affiliations:** 1 Department of Neurology, University of Louisville, Louisville, Kentucky, United States of America; 2 Department of Molecular, Cellular, and Developmental Biology, University of Michigan, Ann Arbor, Michigan, United States of America; Stony Brook University, UNITED STATES

## Abstract

It has become apparent that the intestinal microbiota orchestrates important aspects of our metabolism, immunity, and development. Recent work has demonstrated that the microbiota also influences brain function in healthy and diseased individuals. Of great interest are reports that intestinal bacteria play a role in the pathogenic cascade of both Parkinson and Alzheimer diseases. These neurodegenerative disorders both involve misfolding of endogenous proteins that spreads from one region of the body to another in a manner analogous to prions. The mechanisms of how the microbiota influences or is correlated with disease require elaboration. Microbial proteins or metabolites may influence neurodegeneration through the promotion of amyloid formation by human proteins or by enhancing inflammatory responses to endogenous neuronal amyloids. We review the current knowledge concerning bacterial amyloids and their potential to influence cerebral amyloid aggregation and neuroinflammation. We propose the term “mapranosis” to describe the process of microbiota-associated proteopathy and neuroinflammation. The study of amyloid proteins made by the microbiota and their influence on health and disease is in its infancy. This is a promising area for therapeutic intervention because there are many ways to alter our microbial partners and their products, including amyloid proteins.

Neurodegenerative disorders remain a significant challenge for modern medicine and science. Alzheimer disease, Parkinson disease, amyotrophic lateral sclerosis, and frontotemporal lobar degeneration—as well as the less common disorders progressive supranuclear palsy, corticobasal degeneration, and multisystem atrophy—all have common features. These diseases all have sporadic forms that are responsible for ≥90% of cases [1]. The hallmark of these neurological disorders is the misfolding, aggregation, and deposition of proteins in the brain [2]. Although the misfolded and aggregated proteins are unique to each disorder, the biophysical properties of the aggregates are conserved [2, 3]. The misfolded proteins adopt an ordered amyloid polymer structure with prion-like properties [2], which is associated with sterile cerebral inflammation.

While certain genetic and environmental factors have been implicated in influencing the risk of amyloid-related disorders, the causes remain largely unknown. The field has been heavily influenced by work showing that proteins associated with neurodegeneration such as amyloid-beta (Aß), alpha-synuclein, tau, and transactive response (TAR) DNA-binding protein 43 all adopt pathogenic cross–beta-sheet structures. Furthermore, these structures may be transmitted between hosts and from one region of the brain to another in a manner similar to prions [2]. This pathogenic spread hypothesis has received substantial experimental evidence, but the initial event in the process is unknown [4–6]. While Prusiner has proposed that the primary misfolding event is stochastic [5], it has been suggested that the misfolding process associated with Parkinson disease originates in the gut [7, 8].

Over the past 10 years, a remarkable series of studies has clearly demonstrated the role of a new influence on the brain: that of our microbiota [9]. We are home to trillions of microbes residing inside us and on all of our surfaces, with the largest concentration found in the gut [10]. Our partner organisms are composed of bacteria, fungi, viruses, archaea, and parasites with whom we have coevolved; their presence in our bodies is not accidental nor optional. Most multicellular organisms on the planet have their own microbiome [11–14]. The human microbiota is an imperative part of our metabolism, digestion, nutrition, development, immunity, and protection from pathogens [15–17]. The functions of the microbiota in immunity are best understood through an evolutionary lens: it is in the adaptive interest of our microbes to maintain a state in which the host tolerates their colonization. It is also in the interest of the host to maintain immune tolerance because the microbes contribute to our survival [15–17]. Furthermore, because of evolutionary conservation of molecular structures, exposure to the microbiome and its products provides an excellent opportunity for disease to be created through mechanisms of molecular mimicry [8]. The several influences of the microbiota on the brain have now been established and a new concept of the gut–brain axis delineated (for review see [9].)

The intestinal microbiota presents a dynamic living biome that presents a massive potential threat requiring that a continuously vigilant state of surveillance be maintained. The surveillance involves the sampling of intestinal content by epithelial microfold M cells, as well as dendritic cells, to convey antigens to immune cells in Peyer patches and other intestinal lymphoid elements [18, 19] (Fig 1). Goblet cell–associated antigen passages (GAPS) are another route by which antigens may access the immune system [20]. Moreover, it was recently found that enteroendocrine cells, which are part of the gut epithelium, have properties of neurons and connect directly to alpha-synuclein–containing nerves. It is also critical to recall that the gut has a rich innervation, and there are more neurons in the gut than in the spinal cord [21]. The bidirectional neuronal pathways between gut and brain provide a mode of entry of agents into the brain from the microbiota that bypasses the circulatory system. This is one mode of entry of prions into the central nervous system (CNS) [22] that may be involved in the neurodegenerative disorders as well. Considerable recent evidence suggests that the agent(s) triggering Parkinson disease reside(s) in the microbiota: constipation is an early feature, and alpha-synuclein deposits are found in gut neurons in the disease. In addition, gut entry of agents triggering protein misfolding is the current understanding of the pathogenesis of bovine spongiform encephalopathy and kuru [22]. If the gut is the site of origin of neurodegenerative disease pathogenesis and the gut home to the majority of the human microbiota, are there specific microbial factors responsible for triggering disease?

**Fig 1:**
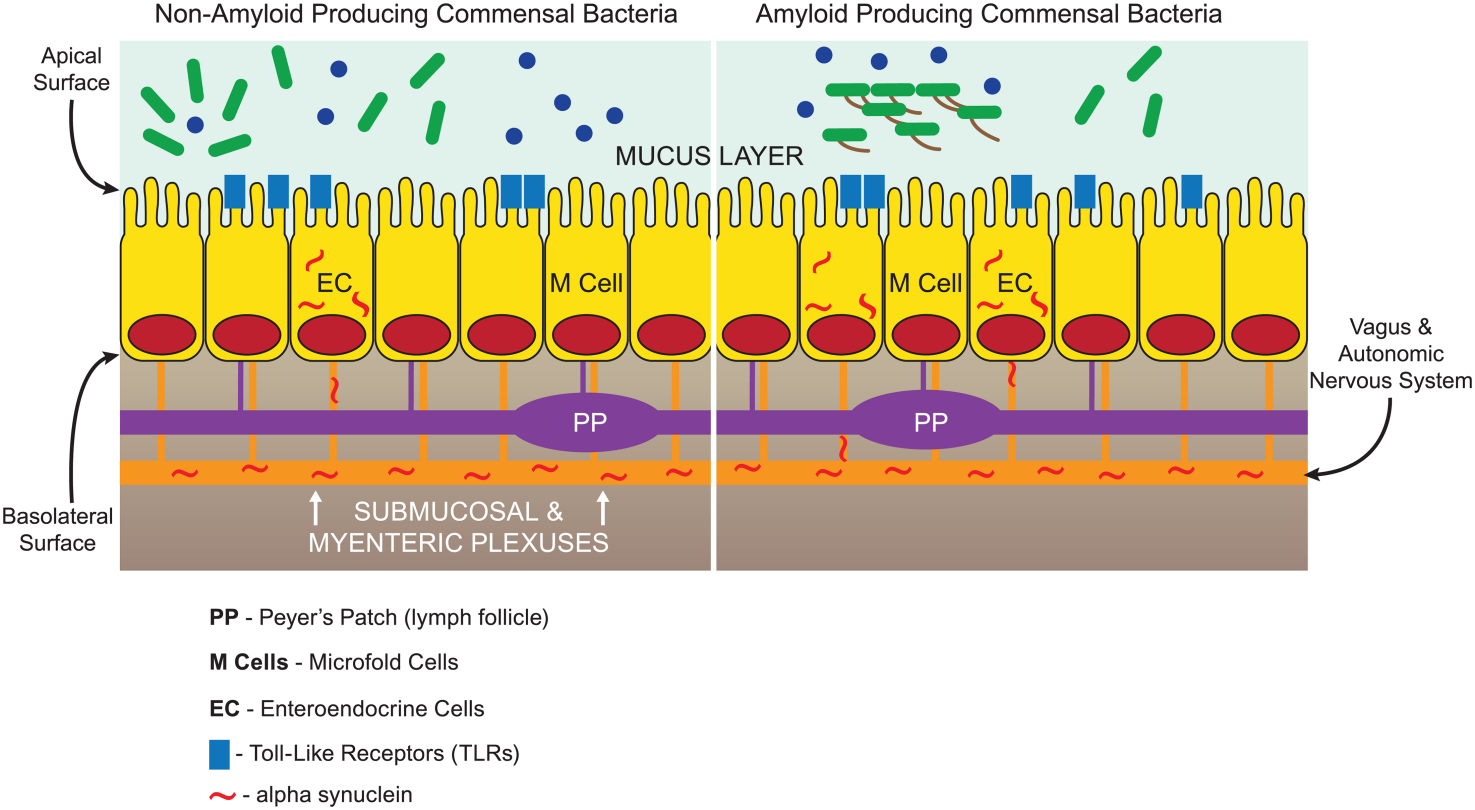
Potential areas of interaction between amyloid-producing bacteria and the gut. Microbial amyloid can engage TLRs on the epithelial surface and prime systemic inflammation through the lymph follicles (Peyer patches) linked to M cells. This priming of the innate immune system via a hematogenous route may cause enhanced response to neuronal amyloids in the brain. Microbial amyloid may also increase production of neuronal amyloids (such as alpha-synuclein) though the neural connections of the enteroendocrine cells as well as the other epithelial cells. Neuronal amyloid accumulation may be enhanced by exposure to microbial amyloid through increased expression and through cross-seeding, leading to misfolding of neuronal proteins in the brain, in a manner analogous to kuru and bovine spongiform encephalopathy. The submucosal and myenteric plexuses are shown as a single structure for simplicity. TLRs, toll-like receptors.

Persons with Parkinson disease have altered bacterial populations [23–27]. The nonuniformity of these results concerning the microbiota in the disease is not surprising, considering dietary and methodological differences [26]. The finding that gut microbiota dysbiosis is observed in patients effected by neurodegenerative diseases is consistent with earlier observations that alpha-synuclein pathology may originate in gut neurons and that early brain pathology in Parkinson disease is found in the cell bodies of the neurons innervating the gut (the dorsal motor nucleus of the vagus nerve) [7]. Furthermore, subjects with cognitive impairment and brain amyloidosis as determined by amyloid imaging have been reported to be colonized by more proinflammatory gut microbes as compared with controls [28]. These results certainly suggest that there is a relationship between the gut microbiota and the genesis of neurological disorders. As this field advances, it has become necessary to go beyond the cataloging of bacterial populations, as suggested by Sharon et al. [10].

In 2002, Chapman et al. discovered that curli, extracellular fibers produced by *Escherichia coli* and other enteric bacteria, share structural and biophysical properties with amyloids [29]. Curli were the first-described amyloid of a new and rapidly growing class of “functional” amyloids [30]. Work has now shown that there are many members of the human microbiome capable of assembling extracellular amyloids, including *Streptococcus*, *Staphylococcus*, *Salmonella*, *Mycobacteria*, *Klebsiella*, *Citrobacter*, and *Bacillus* species. The production of amyloid proteins by *E*. *coli* is highly regulated and functional, as cross–beta-sheet amyloid polymers help bacterial cells bind to one another, form biofilms, and resist destruction by physical or immune agents [31]. Despite a significant growth in our understanding of the production of amyloid proteins in vitro, there have been few studies on the influence of bacterial amyloids in the host.

A noteworthy series of recent papers have also documented a role of the microbiota in neurodegeneration. In 2016, Minter et al. showed that antibiotic exposure lowers gut microbial diversity and ameliorates amyloidosis and neuroinflammation in the APPSWE/PS1ΔE9 double transgenic mouse model of Alzheimer disease [32]. In the same year, Chen et al. reported an investigation of the role that bacterial amyloids may play in alpha-synuclein production and aggregation in aged Fischer 344 rats and *Caenorhabditis elegans*. Rats exposed orally to *E*. *coli*–producing curli were found to have enhanced alpha-synuclein production in the gut and increased production and aggregation of alpha-synuclein in the brain [33]. Increased alpha-synuclein accumulation was accompanied by enhanced cerebral inflammation when compared with animals exposed to identical bacterial strains lacking the ability to produce curli. Cerebral inflammation—with microgliosis, astroglosis, and up-regulation of toll-like receptor (TLR) 2, interleukin 6, and tissue necrosis factor—were also observed in curli-exposed rats. It has been previously shown that microbial amyloids, including curli, may induce immune responses in the intestinal mucosa [34, 35] and regulate the intestinal epithelial barrier, influencing bacterial translocation [36]. Chen et al. also found that transgenic *C*. *elegans* expressing human alpha-synuclein exposed to curli-producing *E*. *coli* had enhanced alpha-synuclein aggregation in muscle, as compared with animals exposed to curli-deficient *E*. *coli* [33].

Sampson and colleagues recently showed that the intestinal microbiota are needed for Lewy body pathology, motor impairment, and microglial activation in alpha-synuclein–overexpressing mice [37]. They found that microbes from Parkinson patients induce more motor impairment in these animals compared with organisms from healthy persons. This work suggests active gut–brain signaling by the microbiota [37]. Furthermore, APPPS1 transgenic mice that overproduce Aß (models of Alzheimer disease) have been found by Harach et al. to harbor altered gut bacteria, as compared with nontransgenic animals [38]. It was found that germ-free Alzheimer disease transgenic mice had reduced pathology, and transfer of microbes from transgenic animals reproduced cerebral Aß findings, but colonization from wild-type mice was less able to enhance cerebral changes.

Bacterial and other amyloid proteins may cross-seed amyloid formation by neuronal proteins [8, 33]. Prions—infectious amyloids—propagate through self-seeding in which a protein in a prion conformation causes another molecule of the same protein to adopt an amyloid conformation [39]. There are many human proteins whose misfolding into amyloid is associated with neurodegeneration. Disease-associated amyloid formation can be supported by self-seeding, which accelerates the early kinetic stage of amyloidogenesis. The nature of prion seeding has been an area of intense study that has revealed the existence of a species barrier in prion propagation (i.e., prions from sheep are unable to induce prion formation in humans, and vice versa). There are, however, several examples of amyloids capable of cross-seeding each other [40–44]. Curli proteins from different bacterial species are capable of cross-seeding amyloid formation both in vitro and in vivo [45, 46]. Lundmark et al. showed that exposure to curli fibrils accelerates serum amyloid A amyloidosis in mice and suggested that curli may serve as a template for fibril formation in vivo [45]. Furthermore, exposure to microbial amyloids in the gut may enhance cerebral nucleation of Aß aggregates [33]. It has been demonstrated that disease proteins, such as alpha-synuclein or Aß, may have strain specificity in a manner analogous to the strains of prion disease [47]. Various bacterial amyloids may elicit cross-seeding in a strain-specific manner, which could account for the various phenotypes of the neurodegenerative disorders. Although cross-seeding has been documented in the lab, we know little about the occurrence of cross-seeding in vivo.

The cross-seeding of amyloid misfolding of alpha-synuclein and other related proteins may access the brain directly through the autonomic nervous system in a manner similar to that established for prion disease. For the cross-seeding mechanism to be involved, it is not necessary for the microbial amyloid proteins to enter the brain themselves. Other pathogenic factors originating from the gut in response to influences of the microbiota include cytokines, lymphokines, lipopolysaccharides, circulating immune cells, and hormones that may be delivered to the brain via neural or hematogenous routes [48]. Access to the brain may also be obtained directly through the nose because the olfactory bipolar cells reside outside the skull in close proximity to the microbes in the roof of the nose. The nasal mucosa has been largely ignored as an important regulatory site for CNS immune system homeostasis [49], and the organisms residing there have just begun to be investigated [50]. Oral bacteria may also make amyloid proteins, including the common oral symbiont *Streptococcus mutans* [51], and oral bacteria have been linked to stroke and cerebral microbleeds (Fig 2) [52].

**Fig 2:**
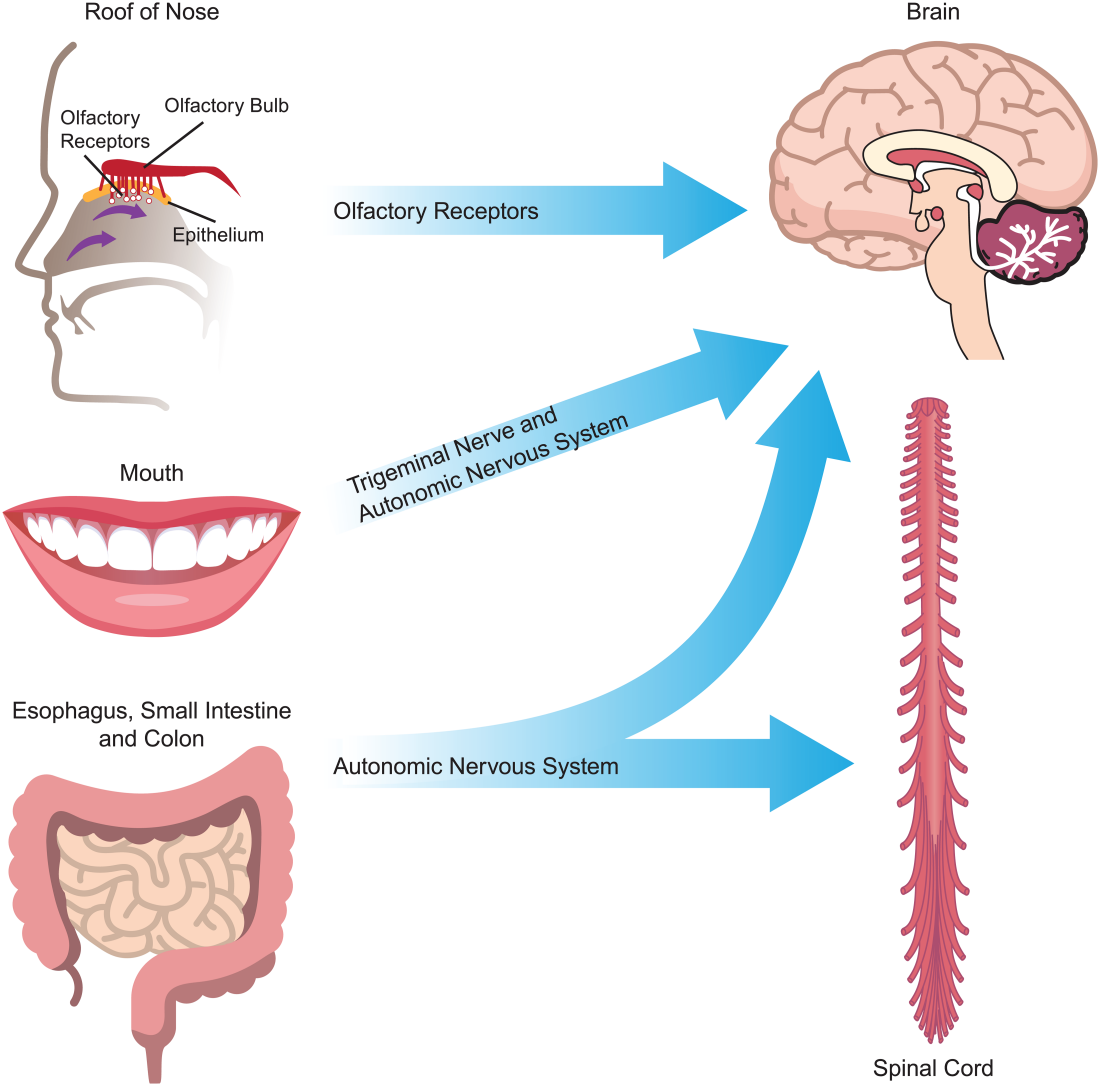
Neural routes by which microbial amyloid may influence the CNS. Microbial amyloid may effect changes in protein folding and neuroinflammation in the CNS through the autonomic nervous system (particularly the vagus nerve), the trigeminal nerve in the mouth and nasopharynx, and the gut (including mouth, esophagus, stomach and intestines), as well as via the olfactory receptors in the roof of the nose. CNS, central nervous system.

In order to enhance understanding of the influence of microbial amyloids on the body, we propose a new term, “MAPRANOSIS.” This refers to the process (“osis”) of microbiota-associated proteopathy and neuroinflammation. We refer to “microbiota” instead of “bacteria” because of the production of fungal and perhaps other nonbacterial microbial amyloids [53, 54]. Proteopathy refers to disease processes involving altered protein structures, which includes amyloid misfolding and other configurations. Although we refer to neuroinflammation in the context of this review, we recognize that other forms of inflammation may also be influenced by microbial amyloids.

In addition to amyloid cross-seeding between bacterial and human amyloidogenic proteins, we must also consider alternative possible mechanisms by which microbial amyloids and other products of the microbiota could influence neurodegenerative disorders.

## 1. The microbiota modulates immunological processes in the CNS [9, 48]

Gut bacteria have been shown to influence microglia in the brain [55], and it has been suggested that the microbiota in Parkinson disease is linked to a higher potential for inflammation [26]. Of interest is the observation that bacterial amyloid is recognized by the innate immune system as a pathogen-associated molecular pattern [56, 57], with a response involving TLRs 2 and 1, cluster of differentiation 14 (CD14), nuclear factor kappa-light-chain-enhancer of activated B cells (NFкB), and inducible nitric oxide synthase (iNOS) [56]. This pathway of immune response is also involved in the recognition of misfolded alpha-synuclein or Aß [58, 59]. Increased expression of TLRs 2 and 4 has been reported on peripheral blood mononuclear cells in Alzheimer disease [60]. Furthermore, microglial cell expression of CD14 is involved in Aß clearance [48], and innate immune system activation by bacterial amyloid may alter Aß homeostasis in the brain.

It has been suggested that exposure to bacterial amyloid proteins in the gut may cause priming of the immune system, thereby enhancing immune response to endogenous production of neuronal amyloids in the brain [8]. This proposal was supported by the work of Chen et al., who found that animals exposed to bacteria producing the amyloid protein curli had higher levels of cerebral inflammation, as compared with animals exposed to bacteria without the ability to produce curli [33]. Neuroinflammation may be a key element in causing the disease phenotype because many older subjects with cerebral amyloid deposits as evaluated by positron emission tomography or autopsy are not cognitively impaired [61]. Inflammatory responses to the amyloid deposits in the brain may be influenced by the microbiota, and this influence may be key to producing cerebral dysfunction [8].

## 2. The microbiota may induce oxidative toxicity and inflammation that contribute to neurodegeneration

Activation of TLRs 2 and 1 by bacterial amyloid is expected to enhance production of free radicals through pathways involving CD14, NFкB, iNOS, and proinflammatory microRNAs [62]. Curli have been shown to activate iNOS [57, 63] as well as NFкB [56]. Rapinski et al. have shown that curli fibers from *Salmonella typhimurium* bind to CD14, an adaptor molecule for the TLR2-TLR1 complex [64]. The influence of the microbiota on the balance of oxidative and/or reductive processes has only recently begun to be investigated [65].

## 3. Metabolites produced by the microbiome may be pathogenic or salutogenic (health sustaining)

Sampson et al. proposed a role for short-chain fatty acids (SCFAs) produced by bacterial fermentation in production of alpha-synuclein–mediated neuroinflammation [37]. And Unger et al. have found a decrease in the concentrations of fecal SCFA in Parkinson subjects [27]. Further work in this area is clearly needed because microbial metabolites, such as SCFAs, may pass the blood–brain barrier and have been shown to have important influences on systemic immunity [66, 67]. Other metabolites that may be of interest include hydrogen sulfide, trimethylamine, neurotransmitters, lipopolysaccharide, bacterial lipoproteins, flagellin, and organic acids [68]. Hydrogen sulfide can be a free radical donor or scavenger, and trimethylamine has been linked to vascular disease, which is involved in Alzheimer disease as well as other dementias [68]. There are further metabolites that await discovery because less than 20% of the genes of our microbiota have a known specific metabolic function [69].

## 4. There is a complex interplay between the human host and its resident microbiota

The influence of host genes on the microbiota may involve dietary choices, bile secretion, and mucus barrier composition, as well as immunological pathways. It is now known that genetic influences on the innate as well as the adaptive immune systems and other pathways influence microbial populations [70, 71]. Cultural differences in human populations can also have a profound influence on the microbiota, including birth practices, child-rearing, food preparation, personal hygiene practices, and diet. Where humans live can influence the composition of the microbiota and thus neurodegeneration. Interestingly, several investigations have reported that both Alzheimer disease and Parkinson disease have a lower age-specific prevalence in Africa and India than in North America or Europe [72]. This may be related to dietary differences, with the higher consumption of vegetables and lower consumption of meat in Africa and India being related to higher levels of fiber intake and production of SCFAs by the microbiota, which enhances the creation of regulatory lymphocytes in the intestine that have anti-inflammatory properties [73]. The crucial influence of global patterns of diet on the microbiota has been demonstrated in a novel study of colon cancer [74]. Collectively, future studies should strive to look beyond the CNS to comprehend the multifaceted factors responsible for brain disorders associated with aging.

Several other questions are apparent concerning the potential role of microbial amyloids and other products of the microbiota on brain disease.

## Why are the neurodegenerative disorders so closely related to age?

Both Alzheimer disease and Parkinson disease are known to have a prolonged course extending for decades from the early to the later stages. It has been documented that the oral transmission of prion disease, such as kuru, may involve an incubation period of >50 years [72]. The enormous size of internal microbial populations and the slow nature of age-related disease processes provide a great opportunity for both pathogenic and salutogenic processes to occur. The late onset of Alzheimer disease and Parkinson disease allows for minute alterations in proteostasis and inflammation to have cumulative effects [75]. It may be that the presence of bacterial amyloids produced by the gut microbiota shortens the time necessary for the development of cerebral amyloids through nucleation of oligomer formation. Also, changes in the immune and gastrointestinal systems with age may enhance the burden of microbial amyloids and alter their influence on immune homeostasis.

## What are the principal sources of microbial amyloid in humans?

The production of bacterial amyloid proteins by organisms known to be found in the gut are well studied [46, 76–79]. However, the metaproteomic study of endogenous microbial amyloids present in the body has not been completed, and we know relatively little about microbial amyloid assembly in the body. It will be necessary to include the production of amyloids by fungal as well as bacterial members [53, 54], and new microbiome studies should consider the taxonomic drivers of microbiota function, including the production of amyloid proteins [80].

## What can be done in regard to prevention and therapy?

The role that intestinal microbes play during Alzheimer disease, Parkinson disease, and related disorders is a particularly dynamic pursuit because the composition and diversity of the microbiota are so variable in the human population. We are just beginning to see a plethora of approaches involving prebiotics, probiotics, antibiotics, dietary interventions, fecal transplants, and other means by which “gene therapy” of microbial nucleotide sequences can be accomplished [81]. Therapeutic approaches to neurodegenerative disease may someday include adapting the gut bacteria to support salutogenic species and other means. We must ask if disease phenotypes can be modulated in persons after the initiation of disease [37]. Although prevention is generally easier than cure, we should not assume a priori that the contribution of the microbiota to neurodegeneration cannot be repaired. The plastic nature of the CNS and the capability of recovery should not be underestimated.

A unique partnership of scientific disciplines is needed to understand the complex interactions of human genes, diet, microbes, and aging. The diversity of bacteria, bacterial metabolites, and dietary fibers and other nutrients has been linked to human health and aging [82]. Developments in the last few thousand years of human history have decreased our microbiome diversity compared with that of our ancestors, with arguably negative consequences for our health [83]. In addition to recognizing the importance of this biological diversity, we need also to be aware of the need for a diversity of approaches to these multifaceted problems. It is not likely that investigators working with a singular approach will provide both the questions and the answers we need. The multidisciplinary nature of the processes by which our partner organisms influence our health suggests that we realize how little we know and recall the saying of James Clerk Maxwell, “Thoroughly conscious ignorance is the prelude to every real advance in science.”
